# Effect of adenosine on short‐term synaptic plasticity in mouse piriform cortex in vitro: adenosine acts as a high‐pass filter

**DOI:** 10.14814/phy2.13992

**Published:** 2019-02-10

**Authors:** Simon P. Perrier, Marie Gleizes, Caroline Fonta, Lionel G. Nowak

**Affiliations:** ^1^ CerCo Université Toulouse 3 CNRS Toulouse Cedex France

**Keywords:** A1 receptor, oscillation, piriform cortex, presynaptic inhibition, short‐term plasticity

## Abstract

We examined the effect of adenosine and of adenosine A1 receptor blockage on short‐term synaptic plasticity in slices of adult mouse anterior piriform cortex maintained in vitro in an in vivo‐like ACSF. Extracellular recording of postsynaptic responses was performed in layer 1a while repeated electrical stimulation (5‐pulse‐trains, frequency between 3.125 and 100 Hz) was applied to the lateral olfactory tract. Our stimulation protocol was aimed at covering the frequency range of oscillatory activities observed in the olfactory bulb in vivo. In control condition, postsynaptic response amplitude showed a large enhancement for stimulation frequencies in the beta and gamma frequency range. A phenomenological model of short‐term synaptic plasticity fitted to the data suggests that this frequency‐dependent enhancement can be explained by the interplay between a short‐term facilitation mechanism and two short‐term depression mechanisms, with fast and slow recovery time constants. In the presence of adenosine, response amplitude evoked by low‐frequency stimulation decreased in a dose‐dependent manner (*IC_50_* = 70 *μ*mol/L). Yet short‐term plasticity became more dominated by facilitation and less influenced by depression. Both changes compensated for the initial decrease in response amplitude in a way that depended on stimulation frequency: compensation was strongest at high frequency, up to restoring response amplitudes to values similar to those measured in control condition. The model suggested that the main effects of adenosine were to decrease neurotransmitter release probability and to attenuate short‐term depression mechanisms. Overall, these results suggest that adenosine does not merely inhibit neuronal activity but acts in a more subtle, frequency‐dependent manner.

## Introduction

Neuronal activity in the brain is associated with various oscillatory phenomena. Different kinds of oscillations have been categorized with respect to their frequency ranges and to their associations with vegetative, perceptive or cognitive processes. A slow rhythm (<1 Hz), delta oscillations (1–4 Hz), and spindles (11–15 Hz) are characteristics of non‐REM sleep (Adrian and Matthews 1934; Steriade et al. 1993; McCormick et al. 2015). Alpha waves (8–12 Hz) were initially observed during quite rest with eyes closed (Berger 1929) and have later been shown to be modulated by attentional processes (reviewed in: Palva and Palva 2007; VanRullen et al. 2014). Beta oscillations (13–35 Hz) are mainly associated with motor preparation (Jasper and Penfield 1949; reviewed in: Kilavik et al. 2013). Gamma band fluctuations (>35 Hz) have been abundantly studied in sensory integration domain (Chatrian et al. 1960; reviewed in: Engel et al. 1997; Gray 1999) and are also associated with a variety of cognitive processes (reviewed in: Tallon‐Baudry 2012; Bosman et al. 2014). The functional meanings and consequences of oscillations are currently little understood (e.g., Shadlen and Movshon 1999; Merker 2013). Our hypothesis is that oscillatory activities dynamically regulate information flow through short‐term synaptic plasticity.

Short‐term synaptic plasticity (STP) refers to the modulation of synaptic efficacy that takes place on a fast‐time scale (few msec to few minutes). Increase or decrease in synaptic response amplitude is referred to as short‐term facilitation (STF) and short‐term depression (STD), respectively. Both facilitation and depression rest on several mechanisms, usually presynaptic and with different time scales (reviewed in: Zucker and Regehr 2002; Fioravante and Regehr 2011; Hennig 2013; de Jong and Fioravante 2014).

In a previous in vitro study (Gleizes et al. 2017), we studied STP in the adult mouse olfactory system, at the connection between the olfactory bulb output and layer 1a of the anterior piriform cortex. In the olfactory bulb, odorant stimulation triggers three kinds of oscillations: beta (15 to 35–40 Hz) and gamma (>35–40 Hz) fluctuations that are superimposed onto a slow breathing rhythm (1–10 Hz) (e.g., Adrian 1950; Chapman et al. 1998; Buonviso et al. 2003; Neville and Haberly 2003; Wesson et al. 2008; Fourcaud‐Trocmé et al. 2014). The prevalence of beta or gamma oscillations has been shown to be modulated by the behavioral context and tasks (e.g., Martin et al. 2004; Beshel et al. 2007). The firing of action potentials by olfactory bulb projection neurons (mitral and tufted cells) has been shown to be phase‐locked with both beta‐ and gamma‐band oscillations (Gray and Skinner 1988; Eeckman and Freeman 1990; Kashiwadani et al. 1999; Cenier et al. 2009; Fourcaud‐Trocmé et al. 2014). Efferent axons from olfactory bulb projection neurons form the lateral olfactory tract (LOT). The LOT hence conveys these rhythmic activities to several brain regions (Price 1973; Haberly and Price 1977), of which the anterior piriform cortex is the largest. Our study showed that, provided extracellular calcium is at physiological concentration, STP leads to a strong enhancement of the postsynaptic response amplitude, especially when elicited at frequencies corresponding to beta and gamma oscillations. A phenomenological model fitted to the data suggested that the frequency‐dependence of this enhancement was determined by the interaction between STF and STD mechanisms acting on different time scales (Gleizes et al. 2017).

Using the same approach, we focused here on the effects of adenosine on STP. Adenosine is a ubiquitous molecule with multiple physiological functions. Originally described as a powerful vasodilator (Drury and Szent‐Györgyi 1929; Gillespie 1934), adenosine has later been shown to possess antinociceptive (Post 1984; Johansson et al. 2001), neuroprotective (Dunwiddie and Masino 2001; Johansson et al. 2001; Gomes et al. 2011), anticonvulsive (Maitre et al. 1974; Dragunow et al. 1985; Fedele et al. 2006; Boison 2016) and hypnogenic (Haulică et al. 1973; Porkka‐Heiskanen et al. 2000; Fredholm et al. 2005) actions. Adenosine may also be involved in the etiology of multiple diseases including epilepsy, Alzheimer's disease, or Parkinson's disease (Fredholm et al. 2005; Gomes et al. 2011; Boison 2016).

In the brain, adenosine effects result in a large part from extracellular adenosine acting as a neuromodulator (Cunha 2001; Dunwiddie and Masino 2001). Extracellular adenosine concentration increases as a consequence of increases in neuronal activity (e.g., Pull and McIlwain 1972; Cunha et al. 1996; Parkinson and Xiong 2004; Wall and Dale 2013). Extracellular adenosine is produced through two mechanisms: first, neuronal activity may lead to an increase in intracellular adenosine, which is externalized through nucleotide transporters (NTs) (reviewed in: King et al. 2006). The degradation of ATP released by neurons and/or glial cells provides a second source of extracellular adenosine. Extracellular ATP is catabolized in ADP, AMP, and adenosine by four families of ecto‐nucleotidases called ecto‐nucleoside triphosphate diphosphohydrolases, ecto‐nucleotide pyrophosphatase/phosphodiesterases, ecto‐5′‐nucleotidase, and tissue nonspecific alkaline phosphatase (e.g., Zimmermann et al. 2012).

Extracellular adenosine has a depressant action on neuronal activity in many cerebral regions. In cortical regions, this is mostly due to the activation of adenosine A1 receptors (e.g., Reddington et al. 1982; Collins and Anson 1985; Fontanez and Porter 2006; reviewed in: Cunha 2001; Dunwiddie and Masino 2001). A1 receptors are to be found over all neuronal compartments, in particular on synaptic terminals of excitatory neurons (Goodman et al. 1983; Tetzlaff et al. 1987) where they produce presynaptic inhibition by reducing presynaptic calcium currents (Hamilton and Smith 1991; Wheeler et al. 1994; Wu and Saggau 1994; Emptage et al. 2001). As an increase in neuronal activity leads to an increase in extracellular adenosine concentration, adenosine acting through A1 receptors thus provides a negative feedback by reducing synaptic transmission. Hence, extracellular adenosine contributes to couple cell metabolism to synaptic transmission.

The question arises then, as to how synaptic transmission between neurons that fire according to diverse brain rhythms would be modified in the presence of adenosine. Using a stimulation protocol adapted to study consequences of oscillations on signal transmission, we explored the effects of adenosine on STP at the LOT‐layer 1a synapse of the anterior piriform cortex. To quantify STP mechanism parameters, we relied on a model adapted from that of Tsodyks et al. (1998) and Oswald and Urban (2012). Our results show that adenosine had two opposite actions: on one hand, adenosine reduced the amplitude of the postsynaptic responses when these were elicited at low frequency, as documented previously (Kuroda et al. 1976; Scholfield 1978; Okada and Saito 1979; Collins and Anson 1985; McCabe and Scholfield 1985; Yang et al. 2007). On the other hand, adenosine application resulted in stronger apparent STF, especially for stimuli delivered at high frequency, to the extent that it could cancel out initial response reduction. This implicates that the action of adenosine is frequency‐dependent, resulting in a stronger attenuation for low frequency inputs than high‐frequency inputs.

## Materials and Methods

### Ethical approval

All procedures were conducted in accordance with the guidelines from the French Ministry of Agriculture (décret 87/848) and from the European Community (directive 86/609) and were approved by the local ethics committee (comité d’éthique Midi‐Pyrénées pour l'expérimentation animale, N° MP/06/79/11/12).

### Brain slice preparation and ACSF composition

Brain slices were prepared as previously described (Gleizes et al. 2017). In short: the brains of 2‐ to 4‐month‐old C57BL/6 female mice were extracted after deep anesthetization with isoflurane. Brain extraction and brain slicing were performed in an ice‐cold, oxygenated (95% O_2_/5% CO_2_), high‐magnesium/calcium free ACSF of the following composition (mmol/L): NaCl 124, NaHCO_3_ 26, KCl 3.2, MgSO_4_ 1, NaH_2_PO_4_ 0.5, MgCl_2_ 9, Glucose 10. Four‐hundred‐micrometer thick slices were cut with a vibratome. The slices were cut in the coronal plane with a cutting angle that allowed preserving the axons issued from the LOT and innervating the anterior piriform cortex. After cutting, the slices were fully submerged in a storage chamber in an oxygenated, in vivo‐like ACSF, for at least 1 h at room temperature. The in vivo‐like ACSF composition was based on ionic concentrations measured in rodent interstitial and cerebrospinal fluids in vivo (for references see Gleizes et al. 2017) and consisted in (in mmol/L): NaCl 124, NaHCO_3_ 26, KCl 3.2, MgSO_4_ 1, NaH_2_PO_4_ 0.5, CaCl_2_ 1.1, and glucose 10. This ACSF was continuously bubbled with a 95% O_2_/5% CO_2_ mixture (pH 7.4).

### Stimulation and recording

For recording, a slice was transferred to a submersion‐type chamber continuously perfused with oxygenated in vivo‐like ACSF (flow rate 3–3.5 mL/min). All recordings were performed at 34–35°C. Both electrical stimulation and extracellular LFP recordings were performed through tungsten‐in‐epoxylite microelectrodes (FHC, 0.2–0.3 MΩ). “Sharp” intracellular recordings were performed through glass micropipettes filled with 3 M K‐Acetate (50–90 MΩ). The micropipettes were made from 1.2 mm OD medium‐walled capillaries with filament (GC120F, Harvard Apparatus) pulled on a P97 Flaming Brown puller. Stimulating electrodes were implanted in the LOT. Stimulation consisted in monopolar cathodal square current pulses (200 *μ*sec duration) delivered by an isolated stimulator (A365 stimulus isolator, WPI).

Local field potentials (LFPs) were recorded in layer 1a of the anterior piriform cortex. LFP signal was amplified (×1000) and filtered (0.1 Hz‐10 kHz) with a NeuroLog system (Digitimer, UK). Intracellular recording targeted layer 2 of the anterior piriform cortex. Intracellular voltage was amplified (×10) with an AxoClamp 2B amplifier (Axon Instrument, Foster City, CA). Input resistance was determined using square current pulse (−0.2 to −0.5 nA, 120 msec duration). Intracellular recording data were excluded if the membrane potential was more positive than −60 mV, if the input resistance was <20 MΩ, and if the cells were unable to repetitively fire overshooting action potentials during depolarizing square current pulses lasting 120–300 msec. For both intra‐ and extracellular recordings, 50 Hz noise was filtered‐out with a Humbug system (Quest Scientific, Canada). Signals were digitized at 20 kHz (1401plus or power1401, CED, UK). Signals were visualized and processed using Spike2 software (CED, UK) and user‐written scripts.

The LFPs evoked in layer 1a after LOT stimulation are composed of a fiber volley followed by a slow negative wave (N‐wave) (Yamamoto and McIlwain 1966). The fiber volley reflects the propagation of action potentials in axons synchronously activated by the stimulation while the N‐wave corresponds to the monosynaptic excitatory postsynaptic potentials generated in the vicinity of the recording electrode. N‐wave amplitude and fiber volley amplitude were measured as their peak amplitudes relative to prestimulus baseline. Stimulation intensity impacts on current spread and determines the stimulated volume (Nowak and Bullier 1996). It was kept relatively low (6–40 *μ*A, 200 *μ*sec duration) in an attempt to avoid contamination of the N‐wave by fast positive components likely due to postsynaptic action potentials generation (Richards and Sercombe 1968) and to avoid recruitment of polysynaptic responses.

In order to examine the effect of adenosine on synaptic response amplitude and short‐term synaptic plasticity, five *exogenous* adenosine concentrations have been tested (10, 30, 100, 300 and 1000 *μ*mol/L). In addition, the influence of *endogenous* adenosine was examined using the A1 receptor antagonist 8‐cyclopentyl‐1,3‐dimethylxanthine (CPT) and the A2_A_ receptor antagonist 4‐(‐2‐[7‐amino‐2‐{2‐furyl}{1,2,4}triazolo{2,3‐a} {1,3,5}triazin‐5‐yl‐amino]ethyl)phenol (ZM 241385). Two concentrations of CPT (0.2 and 1 *μ*mol/L) have been used. Since they elicited similar effects, results obtained with the two concentrations have been pooled. Two concentrations of ZM 241385 have been used as well (0.1 and 1 *μ*mol/L). As they both had little effect, results obtained with the two concentrations have been pooled. Adenosine, CPT and ZM 241385 were purchased from Sigma.

### Dose–response relationship

**Figure 1:**
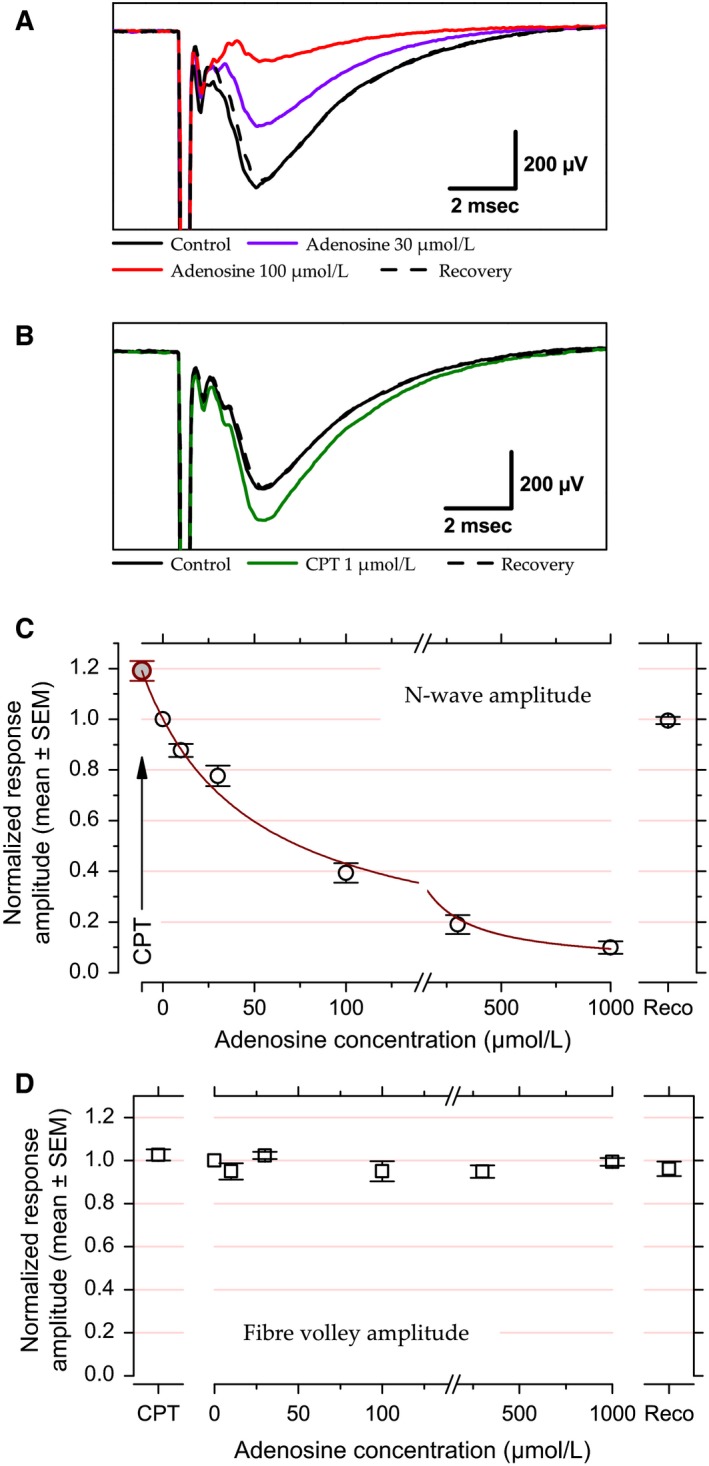
Effects of exogenous and endogenous adenosine on response amplitude evoked at 0.5 Hz at the LOT‐layer 1a synapse. (A) Example of LFP recorded in layer 1a of the piriform cortex with two different adenosine concentrations (30 and 100 *μ*mol/L) compared to control and recovery conditions. (B) Example of LFP recorded in presence or absence of CPT. (C) Population data for N‐wave amplitude fitted with Prince and Stevens (1992)'s model. Data points correspond to the mean and error bars to ±1 SEM computed after normalizing the individual N‐wave amplitudes to their corresponding control values. Red line corresponds to the fit (fit weighted by variance, *R* ^2^ = 0.99). Note that the value obtained in the presence of CPT (arrow, *c*_0_ = −11.4 *μ*mol/L, NRA_max_ = 1.19) was plotted after fitting. (D) Population data for fiber volley amplitude versus CPT and adenosine concentrations. Data points correspond to the mean and error bars to ±1 SEM computed after normalizing the individual fiber volley amplitudes to their corresponding control values. For enhancing data visibility, the *x*‐axis has been split and is presented with different scales before and after the break in C and D.

The effect of adding adenosine or adenosine receptor antagonists on LFP amplitude was monitored using electrical stimulation delivered at 0.5 Hz for 10–20 min. One to three adenosine concentrations were tested between one control and one recovery test. CPT and ZM 241385 were tested individually between one control and one recovery. Controls and recoveries also lasted for 10–20 min. The time‐course of the effects of adenosine, CPT, and ZM 241385 was examined by averaging the data over each consecutive minute (30 responses per average). This analysis revealed that adenosine and CPT exerted their maximal effects within 5 min. The last minute of the series was used for constructing the dose–response relationship (Fig. 1). Data were included only if response amplitude after recovery differed by less than ±15% from that obtained in control.

For population data analysis, the data were normalized prior to averaging, with the normalized response amplitudes, *NRA*, corresponding to the peak amplitude of the N‐wave in a given condition expressed as a fraction of the response amplitude in the control condition. The mean *NRA* as a function of extracellular adenosine concentration, *[Ado]*, was then fitted with Prince and Stevens (1992) model:

$$\text{NRA}={\text{NRA}}_{\mathrm{Min}}+\frac{{\text{NRA}}_{\mathrm{Max}}-{\text{NRA}}_{\mathrm{Min}}}{1+\frac{c_{0}+\left[\text{Ado}\right]}{K_{d}}}$$

where NRA_min_ represents the maximal effect of adenosine (horizontal asymptote), NRA_max_ represents the response amplitude in the presence of CPT, that is, without endogenous adenosine action, *c*_0_ the estimated endogenous adenosine concentration, and *K*_*d*_ the dissociation constant of the complex formed by adenosine and adenosine A1 receptor.

### Short‐term plasticity

The STP protocol was applied after each 0.5 Hz stimulation period. STP was tested using stimulation trains consisting of five consecutive stimuli delivered at six different frequencies: 3.125, 6.25, 12.5, 25, 50, and 100 Hz. Stimulation trains were limited to five successive pulses for two reasons: first because the number of successive cycles in the olfactory bulb oscillation is typically between 4 and 10 (e.g., Buonviso et al. 2003; Neville and Haberly 2003; Fourcaud‐Trocmé et al. 2014), and second, because larger number of pulses may recruit a slow adaptation (Richards 1972; Best and Wilson 2004) that would have complicated our model‐based analysis. Ten seconds without stimulation followed each train to ensure recovery of synaptic resources. Each train was repeated 10 times to allow for averaging. The 10 traces obtained at a given frequency were averaged as a function of pulse ordinal number (1–5).

For population‐level analyses and for model fitting, the *relative response amplitude* (RA_*n*_) was calculated by dividing the amplitude of the N‐wave obtained at the *n*th stimulation pulse (*A*_*n*_ *)* in control as well as in adenosine and in CPT by that obtained at the first pulse (*A*_1_) of each stimulation train in the *control* condition (RA_*n*_ = *A*_*n*_/*A*_1_, *n* between 1 and 5). This normalization by the first response in the control condition was applied to the responses obtained in control as well as in the corresponding adenosine and CPT conditions.

### Short‐term plasticity model

We used a phenomenological model to achieve a quantitative description of the influence of adenosine on STP parameters at the LOT‐layer 1a synapse. This model, initially adapted from that developed by Tsodyks et al. (1998) and Oswald and Urban (2012), has been described previously (Gleizes et al. 2017). Two versions of the model were used in this study: the first stipulates that one single facilitation mechanism can account for the data while the second allows for the presence of either one or two depression mechanisms in addition to facilitation.

In response to the first stimulation of a train, the relative response amplitude is:

$${\text{RA}}_{1}=E\cdot U=1$$

where *E* depicts the global synaptic efficacy and *U*, the utilization of efficacy *E*, which corresponds to the proportion of *E* that is used at the first stimulation. *U* may be conceived as the initial release probability, while *E* would correspond to the theoretical maximal amplitude that would be obtained if the synapses released all their synaptic vesicles and/or if all postsynaptic receptors saturated.

In its full version, the aim of the model was to fit, for each stimulation rank *n *>* *1, RA as the product of four terms:

$${\text{RA}}_{n}=E\cdot r_{1,n}^{-}\cdot r_{2,n}^{-}\cdot u_{n}^{+}$$

where “minus” and “plus” indicate values, respectively, just before the stimulation and at stimulation time. *u*, the utilization of efficacy, implements the amount of facilitation as a fraction of *E*: for a pulse, *u* rises by a fraction of its value at the first pulse, *U*; then, during the interpulse interval (IPI), *u* decays back to zero according to the time constant of facilitation (*τ*_*F*_) as follows:

$$\text{at stimulation time}:\;u_{n+1}^{+}=u_{n+1}^{-}+U\cdot \left(1-u_{n+1}^{-}\right);$$

$$\text{during}\;\text{IPI}:\;u_{n+1}^{-}=u_{n}^{+}\cdot e^{\frac{-\mathrm{IPI}}{\tau_{F}}}$$

*r*_1_ and *r*_2_ represent two reserves of available synaptic resources *E*, such as pools of neurotransmitter vesicles or availability of postsynaptic receptors. *r*_1_ and *r*_2_ are ascribed to two STD mechanisms that are distinguished by their dynamics. At rest, *r*_1_ = *r*_2_ = 1. Then, at each stimulation, utilization of efficacy *u* impacts on both reserves. However, to avoid an equal influence on the two synaptic reserves, *u* is assumed to be distributed by factors *k* and (1 − *k*), respectively:

$$\begin{array}{cc} r_{1,n+1}^{+}= & r_{1,n+1}^{-}-r_{1,n+1}^{-}\cdot u_{n+1}^{+}\cdot k;\\ & {2,n+1}^{+}=r_{2,n+1}^{-}-r_{2,n+1}^{-}\cdot u_{n+1}^{+}\cdot \left(1-k\right) \end{array}$$

During the IPI, *r*_1_ and *r*_2_ recover with time constants that correspond to two distinct time constants of recovery from depression (respectively, *τ*_*R*1_ and *τ*_*R*2_), as follows:

$$\begin{array}{cc} r_{1,n+1}^{-}= & \;\left(r_{1,n}^{+}-1\right)\cdot e^{\frac{-\mathrm{IPI}}{\tau_{R1}}}+1;\\ & {2,n+1}^{-}=\left(r_{2,n}^{+}-1\right)\cdot e^{\frac{-\mathrm{IPI}}{\tau_{R2}}}+1 \end{array}$$

By construction, the first depression mechanism was assumed to be faster than the second one. The second depression mechanism was dismissed when *k *=* *1.

In some cases, neither fast nor slow depressions were required to fit the data and the model was simplified by removing the *r* terms as follows: ${\text{RA}}_{n}=E\cdot u_{n}^{+}$.

The model was adjusted to several datasets at the same time, with *E* as a shared parameter. Datasets corresponded to one control associated with one to three adenosine concentrations, or to one control and one CPT condition issued from the same experiment. Hence, one set of parameters was returned for each control, adenosine or CPT condition except for *E* that was assumed to be constant across experimental conditions. The model fit was optimized by an iterative procedure that minimized the mean‐squared error (MSE) between measured and estimated amplitudes. Nelder and Mead (1965) method was used for determining MSE. During parameter optimization, *E*,* U*,* k*,* τ*_*F*_, *τ*_*R*1_, and *τ*_*R*2_ were constrained as follows: *E*,* U*, and *k* between 0 and, respectively, 10, 1, and 1; *τ*_*F*_, *τ*_*R*1_, and *τ*_*R*2_ between 0 and 3 seconds with the supplementary constraint that *τ*_*R*1_ had to be inferior to *τ*_*R*2_. When optimal *k* was equal to 1, *τ*_*R*2_ had no more influence and was withheld from further analysis. Robustness of fitting was quantified using the root mean‐squared error (RMSE).

### Statistics

Raw population data are summarized by their means ± 1 SEM and comparisons (ratios expressed as percentages) are summarized by their means and 95% confidence intervals (between brackets). Error bars in Figures delimit the SEM. Paired *t*‐test and ANOVA were used for statistical comparisons. When ANOVA was used, post‐hoc comparisons were performed using Fischer's PLSD. When paired *t*‐tests were used, Holm‐Šídák formula was used to correct for multiple testing. *P‐*values given in text are the corrected *P‐*values.

## Results

### Effect of adenosine on response amplitude

Previous studies established that adenosine inhibits synaptic transmission at the LOT‐layer 1a synapse of the piriform cortex (Kuroda et al. 1976; Scholfield 1978; Okada and Saito 1979; Collins and Anson 1985; McCabe and Scholfield 1985; Yang et al. 2007) and that this inhibitory action is mediated by presynaptic adenosine A1 receptor activation (Collins and Anson 1985; McCabe and Scholfield 1985; Yang et al. 2007). We first wished to confirm and quantify adenosine inhibitory effect by recording LFPs in layer 1a of the anterior piriform cortex while the LOT was stimulated at 0.5 Hz, a stimulation frequency that minimally recruited STP mechanisms – Gleizes et al. (2017) reported a 3% difference in amplitude between responses evoked at the beginning and at the end of a 5 min long stimulation train at 0.5 Hz.

Effects of adenosine and of adenosine receptor blockade have been examined in 39 slices from 39 mice (1 slice/mouse). Five different adenosine concentrations have been used: 10 *μ*mol/L (*N* = 6), 30 *μ*mol/L (*N* = 12), 100 *μ*mol/L (*N* = 13), 300 *μ*mol/L (*N* = 10), and 1000 *μ*mol/L (*N* = 5). Adenosine A1 receptor blockade was tested with CPT, a selective A1 adenosine receptor antagonist (Bruns et al. 1986). Two CPT concentrations (0.2 and 1 *μ*mol/L) have been used. As the two concentrations yielded similar results, the data were pooled (*N* = 20). Adenosine A2_A_ receptor blockade was tested with ZM 241385, a selective A2_A_ receptor antagonist (Poucher et al. 1995), in five experiments at 100 nmol/L (*N* = 3) and 1 *μ*mol/L (*N* = 2); results obtained with the two concentrations have been pooled as they appeared similar. The total number of “tests” (one single adenosine concentration or adenosine receptor antagonist concentration) was 71. CPT and ZM 241385 administration was systematically preceded by a control and followed by a recovery, yet between one and three adenosine concentrations were tested between one control and one recovery, such that the total number of controls and recoveries (*N* = 54) is less than the total number of tests.

Figure 1A shows examples of adenosine effects on LFP in layer 1a. Effects of two different adenosine concentrations (30 and 100 μmol/L) are illustrated. Adenosine at 100 *μ*mol/L reduced N‐wave amplitude to about 20% of the control value. Adenosine at 30 *μ*mol/L produced a weaker inhibition, with a response decrease by about −40%.

Previous studies revealed an inhibitory tone exerted by ambient adenosine in piriform cortex (McCabe and Scholfield 1985; Yang et al. 2007). We used CPT to reveal this inhibitory tone. Figure 1B presents an example where CPT (1 *μ*mol/L) led to an increase in the N‐wave amplitude by +20% relative to the control situation.

Population data for N‐wave amplitude are displayed in Figure 1C. Data were normalized to the amplitude in control condition before averaging (see Methods). At the population level, all manipulations, except those with ZM 241385, resulted in significant changes in response amplitude in comparison to control response amplitude (paired *t*‐test, *P *<* *0.004). Response amplitude did not change significantly in the presence of ZM 241385 (*P* = 0.14, +7 ± 4%, not illustrated), suggesting that endogenous adenosine does not influence response amplitude through A2_A_ receptors.

The constancy of fiber volley amplitude in Figure 1A and B suggests that neither adenosine nor CPT affected action potential propagation in axons. Figure 1D illustrates normalized fiber volley amplitude at the population level versus CPT and adenosine at 10–1000 lmol/L. Fiber volley could not be measured in all cases, in particular when it merged with the stimulus artifact. The sample was therefore composed of: control, *N* = 31; adenosine 10 *μ*mol/L, *N* = 3; adenosine 30 *μ*mol/L, *N* = 8; adenosine 100 *μ*mol/L, *N* = 6; adenosine 300 *μ*mol/L, *N* = 7; adenosine 1000 *μ*mol/L, *N* = 5; and CPT, *N* = 12. Fiber volley amplitude was unaffected by adenosine or CPT (*P* ≥ 0.4 for all comparisons, paired *t*‐test). Hence, changes in N‐wave amplitude can be attributed to changes taking place at the synapse.

The normalized N‐wave amplitude as a function of adenosine concentration has been fitted with Prince and Stevens (1992)'s model (see Methods) (Fig. 1C). In the presence of CPT, the mean response amplitude was 19% above the control value. The *x*‐value associated with CPT was estimated at −*c*_0_ with a value of −11.4 *μ*mol/L (note that the data point corresponding to the CPT data in Figure 1C was plotted after fitting). In other words, the endogenous adenosine concentration in our experimental condition is equivalent to 11.4 *μ*mol/L of bath‐applied adenosine. The actual endogenous concentration is likely to be much lower due to the presence of uptake and degradation mechanisms for adenosine (see Discussion). The maximal inhibition (horizontal asymptote) was extrapolated to a response amplitude close to zero (NRA_Min_ = 3%). The adenosine concentration that led to 50% of the maximal inhibition (*IC_50_*) was 70 *μ*mol/L.

Intracellular recordings (not illustrated) have been performed simultaneously with LFP recordings in five cells. Four of these cells displayed monosynaptic EPSPs in response to LOT stimulation. These recordings confirmed that adenosine exerted its inhibitory action at the synaptic level: perfusion of adenosine (100 *μ*mol/L) resulted in a nonsignificant (*P* = 0.19, paired *t*‐test) hyperpolarization by 1.6 ± 1.0 mV. Adenosine also had no effect on input resistance (+0.5%, *P* = 0.6). At the same time, postsynaptic response amplitude was reduced to 25% of control response amplitude in the four cells that displayed monosynaptic EPSPs. Among these four cells, three displayed an intrinsically bursting (IB) phenotype and likely corresponded to superficial pyramidal cells, while the remaining cell displayed a regular spiking (RS) behavior suggesting it was a semilunar cell (Suzuki and Bekkers 2006). It is difficult to make definitive statement on such a small sample but it is noticeable that adenosine decreased the amplitude of the postsynaptic responses in both cell types (19% of control response amplitude in the RS cell, 26 ± 13% in the IB cells).

### Effects of adenosine on short‐term plasticity

**Figure 2:**
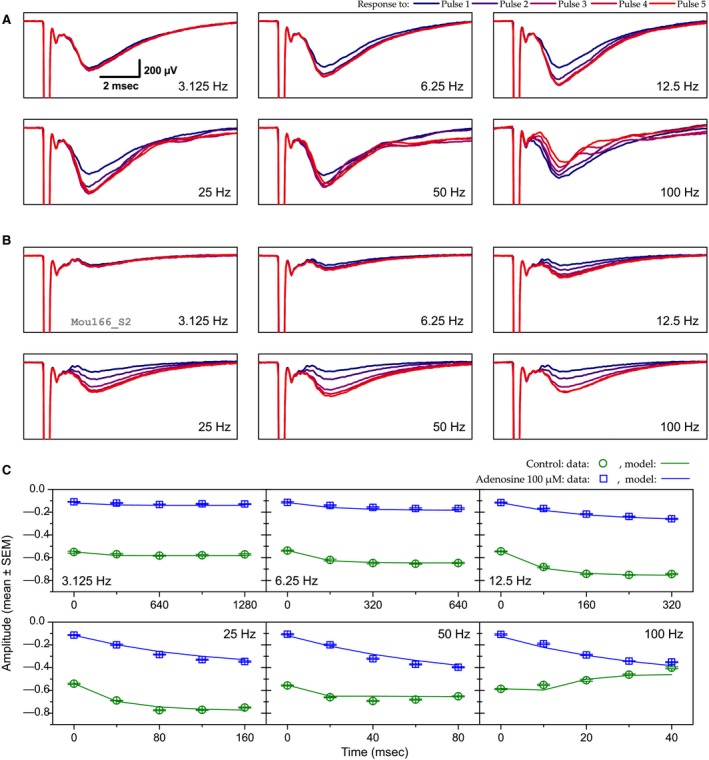
Example of the effect of adenosine (100 *μ*mol/L) on short‐term synaptic plasticity. (A and B) The six panels in A and B correspond to the six stimulation frequencies (from 3.125 to 100 Hz). Each panel shows the mean LFP trace for each of the five consecutive stimuli of a stimulation train at a given frequency. Pulse ranks are color coded from the first one (blue) to the fifth one (red). Results obtained in the control condition and in the presence of adenosine 100 *μ*mol/L are represented in A and B, respectively. Scale in the 3.125 Hz panel in A applies to all other panels. (C) Peak N‐wave amplitude (in mV) as a function of stimulus timing and frequency. Points represent the mean experimental data (error bars denote ±1 SEM) while solid lines represent STP model fits. Green symbols and lines correspond to control situation and blue ones to adenosine 100 *μ*mol/L. Model parameters: shared parameter, *E* = 1.957. Control: *U* = 0.509, *τ*_*F*_ = 151 msec, *k* = 1, *τ*_*R*1_ = 19 msec; adenosine 100 *μ*mol/L: *U* = 0.11; *τ*_*F*_ = 184 msec; *k* = 1; *τ*_*R*1_ = 11 msec. RMSE = 0.032.

**Figure 3:**
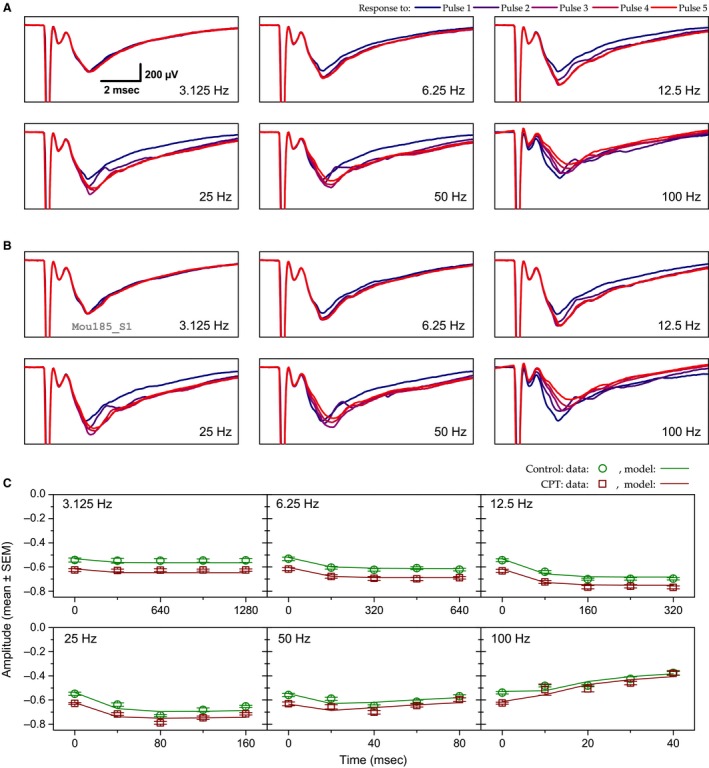
Example of CPT effect on short‐term synaptic plasticity. Same conventions as in Figure. 2. (A) control LFPs. (B) LFPs in CPT 1 *μ*mol/L. (C) N‐wave amplitude, experimental data, and model fit. Green symbols and lines: control; red symbols and lines: CPT. Model parameters: shared parameter, *E* = 1.703. Control: *U* = 0.575, *τ*_*F*_ **= 163 msec, *k* = 0.916, *τ*_*R*1_ = 14 msec, *τ*_*R*2_ = 100 msec; CPT 1 *μ*mol/L: *U* = 0.666; *τ*_*F*_ = 167 msec; *k* = 0.89; *τ*_*R*1_ = 12 msec, *τ*_*R*2_ = 62 msec. RMSE = 0.039.

For examining the effect of adenosine on STP at the LOT‐layer 1a synapse, we used a stimulation protocol that consisted in trains of five stimuli delivered at six different frequencies between 3.125 Hz and 100 Hz. Examples of averaged LFPs for each frequency and each pulse ordinal number are presented in Figures 2 and 3. The data illustrate two experiments, one on the effect of adenosine at 100 *μ*mol/L (Fig. 2) and the other on the effect of CPT at 1 *μ*mol/L (Fig. 3). Fiber volleys were stable across successive pulses in a train, allowing to conclude that variations in N‐wave amplitude were not due to variations in axonal transmission with high‐stimulation frequency (lack of significant effect of stimulation frequency on fiber volley amplitude has been documented previously in Gleizes et al. 2017). For both experiments, the peak amplitude of the N‐wave was extracted and was represented as a function of stimulus time in Figures 2C and 3C.

Results for both control conditions (Figs. 2A and C and 3A and C) appear similar. Response amplitude remained fairly constant when stimuli were delivered at 3.125 Hz. With stimulation train between 6.25 and 25 Hz, N‐wave amplitude was progressively enhanced in proportion to the stimulation frequency. The maximal amplitude was reached with the third pulse of the train at 25 Hz (×1.4 relative to the first‐response amplitude in Fig. 2A, ×1.3 in Fig. 3A). Response enhancement was less pronounced at 50 Hz than at 25 Hz and it was no longer visible at 100 Hz. Instead at 100 Hz response amplitude declined during the stimulation train to reach a value representing 68% (Fig. 2A) or 70% (Fig. 3A) of first pulse amplitude at the end of the train.

The addition of 100 *μ*mol/L of adenosine (Fig. 2B and C) resulted in a strong reduction (−80%) of first pulse response amplitudes. Response enhancement during the stimulation train was visible with all frequencies ≥6.25 Hz, including 100 Hz. Maximal enhancement was reached at 50 Hz and was much larger than the maximal enhancement reached at 25 Hz in control condition (×3.75 relative to first pulse amplitude vs. ×1.4). It is noticeable that, in spite of the initial inhibition produced by adenosine, response enhancement at 100 Hz was such that the response amplitude at the end of the stimulation train was very close to that observed in the control situation (Fig. 2C).

When the action of ambient adenosine was prevented with CPT, response amplitude to the first stimulation pulse became larger than in control condition (+15%) (Fig. 3B and C). This difference remained approximately constant for all the stimuli with the three lowest frequencies. Yet for frequencies between 25 and 100 Hz, response amplitude differences progressively weakened; at 100 Hz the initial difference vanished during the stimulation train, as if response decline was stronger in the presence of CPT.

**Figure 4:**
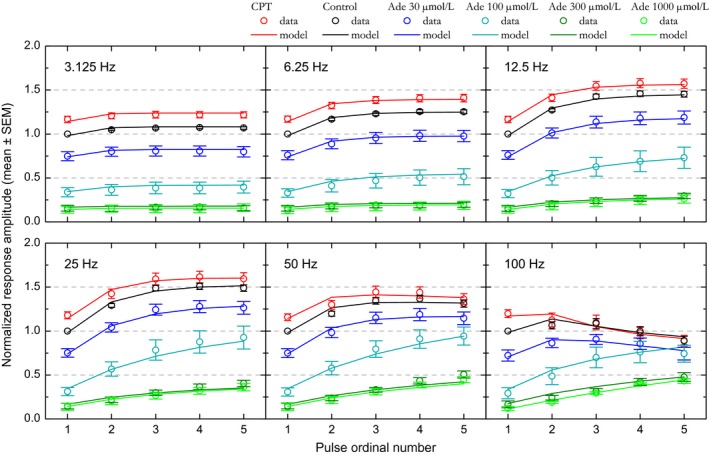
Effect of adenosine and CPT on short‐term synaptic plasticity at the population level. CPT (adenosine A1 receptor antagonist) and different extracellular adenosine concentrations (30, 100, 300, and 1000 *μ*mol/L) have been tested. Relative amplitudes associated with each condition are plotted as a function of stimulation pulse rank and of stimulation frequency. Experimental data are represented by the colored dots. Error bars represent the SEM. The means of the values predicted by the STP model are represented by colored solid lines. SEM values for model predictions were similar to those for experimental data and are not shown for alleviating the figure.

The examples presented in Figures 2 and 3 are representative of the mean observations. STP was examined in datasets consisting in one control and one CPT condition, or in one control and 1–3 adenosine conditions. STP has not been examined in 8 of the 54 datasets used in the dose–response relationship (three cases with adenosine 100 *μ*mol/L and the five cases with ZM241385). Seven datasets have been excluded from the sample due to poor STP model fit (see below). The remaining 39 datasets include the following condition distribution: control: *N* = 39; CPT: *N* = 16; adenosine 10 *μ*mol/L: *N* = 5; adenosine 30 *μ*mol/L: *N* = 12; adenosine 100 *μ*mol/L: *N* = 8; adenosine 300 *μ*mol/L: *N* = 9; adenosine 1000 *μ*mol/L: *N* = 5. Independently of stimulation frequency and pulse ordinal number, response amplitudes were strongly affected by both adenosine and CPT (ANOVA, *P *<* *0.0001) with two exceptions: response amplitudes in adenosine 10 *μ*mol/L did not differ significantly from those in control (Fischer's PLSD, *P* = 0.5) and the results obtained with adenosine at 10 *μ*mol/L are not presented further. Likewise, response amplitudes in adenosine 300 *μ*mol/L did not differ significantly from those in adenosine 1000 *μ*mol/L (*P* = 0.4). Additional ANOVA test using both stimulation frequency and experimental manipulation as independent variables revealed a significant effect of stimulation frequency on response amplitude (*P *<* *0.0001) as well as a significant interaction between stimulation frequency and experimental manipulation (*P *<* *0.0001). This significant interaction indicates that the action of CPT and adenosine on response amplitude depended on stimulation frequency, as illustrated in Figure 4.

The dots in Figure 4 represent experimental data at the population level. Before averaging, the data obtained in control as well as in adenosine and CPT were normalized with respect to the first‐response amplitude measured in the *control* condition. Relative amplitudes are plotted with colored dots, one color per condition.

In control condition (black dots in Fig. 4), stimulation at 3.125 Hz induced a weak (+6% to +7%) yet significant (ANOVA, *P *<* *0.0001) increase in relative amplitudes. Amplitudes plateaued after the second pulse as amplitudes obtained with the second, third, fourth, and fifth stimulation did not differ significantly (Fischer's PLSD, *P *>* *0.1). Relative amplitudes further increased with increase in stimulation frequency (ANOVA, *P *<* *0.0001) up to 25 Hz, where the strongest enhancement was observed: +51% to +54% relative to pulse 1, reached with the third, fourth, and fifth stimulation. At 50 Hz, responses were still enhanced (ANOVA, *P *<* *0.0001), although significantly less than at 25 Hz (+34 to +39%, pulses 3–5, ANOVA, *P *<* *0.0001). For all frequencies between 6.25 and 50 Hz, amplitudes reached their highest values with the third stimulus and plateaued beyond: amplitudes obtained for each frequency with the third, fourth, and fifth stimulation did not differ significantly (Fischer's PLSD, *P *>* *0.3). In contrast to those obtained at lower frequencies, responses obtained at 100 Hz showed a weak, not significant enhancement with the second and third stimulation; instead, relative amplitudes declined after the third pulse until reaching, at the end of stimulation train, an amplitude significantly inferior to the initial one (−11% relative to pulse 1, Fischer's PLSD, *P *<* *0.02). These results confirm those obtained previously in the same experimental conditions but with different datasets (Gleizes et al. 2017).

In adenosine 30 *μ*mol/L (dark blue dots in Fig. 4), first‐pulse response amplitudes were about 75% of that in control condition. Beyond the first pulse, significant response enhancement was observed for all frequencies between 12 and 50 Hz (ANOVA, *P *<* *0.0001). Amplitudes obtained with the third, fourth, and fifth pulse for each of these frequencies did not differ significantly. As in control condition, relative amplitude enhancement reached a maximum between the third and fifth stimulus at 25 Hz, yet signal modulation was stronger (+61% to +73% relative to first amplitude in adenosine 30 *μ*mol/L vs. +51% to +54% in control condition). Although slightly less pronounced at 50 Hz, this modulation (+53% to +59%) did not differ significantly from that observed at 25 Hz (ANOVA, *P* = 0.1). However, this modulation reached a level higher than in control condition. At 100 Hz, response amplitudes did not differ significantly between pulses (ANOVA, *P* = 0.2). In particular, the response decline observed at the end of 100 Hz stimulation train in control condition was not observed in adenosine 30 *μ*mol/L, such that response amplitudes with the third–fifth pulses reached values comparable to those in the control condition (Fischer's PLSD, *P *>* *0.05).

STP behavior became clearly distinct in adenosine 100 *μ*mol/L (cyan dots in Fig. 4). Relative amplitude was significantly enhanced with successive stimuli for all frequencies above 6.25 Hz, including 100 Hz (ANOVA, *P* ≤ 0.015). Enhancement was maximum and equivalent at 25 Hz and 50 Hz (*P* = 0.9). This modulation was much bigger (+180 to +205%, relative to first pulse amplitude, with the fourth and fifth stimulation) than in control condition and in adenosine 30 *μ*mol/L. Furthermore, the plateau appeared less marked than in previous conditions; at 50 Hz in particular, response amplitude with the fifth pulse was similar to that obtained in 30 *μ*mol/L adenosine (Fischer's PLSD, *P* = 0.2). At 100 Hz, response enhancement persisted, although less than at 25 or 50 Hz. This persistent enhancement was sufficient to lead to a relative amplitude similar to the control one at the end of the stimulation train (fourth and fifth stimulus, Fischer's PLSD, *P *>* *0.06).

With adenosine at 30 and 100 *μ*mol/L, the initial inhibition induced by adenosine seemed to be counteracted by a stronger response enhancement. STP allowed preserving an equivalent synaptic transmission in the highest‐frequency bands. In particular at 100 Hz all response amplitudes converged to the one obtained in the control condition. A trend toward such convergence was also noticeable at 50 Hz.

STP behavior appeared to be identical with 300 and 1000 *μ*mol/L of added adenosine (dark green and green dots in Fig. 4), as if a floor for adenosine effect was reached. Response amplitudes with the first stimulus were at the same level (13–15% of control amplitude) and response growths with the next stimuli were very similar. Response enhancement became significant (ANOVA, *P* ≤ 0.03) for frequencies >6.25 Hz with adenosine 300 *μ*mol/L and >12.5 Hz with adenosine 1 mmol/L. With higher stimulation frequencies and in contrast to the other conditions, response amplitude did not plateau and increased almost linearly instead; amplitudes were significantly different (Fischer's PLSD, *P *<* *0.05) between the third and fourth and between the third and fifth stimuli of the train for most frequencies above 12.5 Hz. The strongest response enhancements were observed at 50 Hz in adenosine 300 *μ*mol/L and at 100 Hz in adenosine 1 mmol/L, reaching values between +240 and +260% of first‐pulse response amplitude. Yet, despite this considerable enhancement, response amplitude at the end of the 100 Hz train remained at 48% of the response amplitude obtained at the end of the 100 Hz train in the control condition.

We used CPT to examine STP in the absence of endogenous adenosine action (red dots in Fig. 4). Antagonist effect was visible at the first pulse with an increase by about +17% relative to control response amplitude. Then, as in the example in Figure 3, relative amplitudes were significantly (ANOVA, *P* ≤ 0.003) enhanced with stimulation frequencies between 6.25 Hz and 50 Hz. Relative amplitude reached comparable maxima at 12.5 and 25 Hz, with a signal modulation of +35 and +37% relative to first pulse response amplitude in CPT. As in control condition, relative response amplitude was significantly larger at 25 Hz compared to 50 Hz (ANOVA, *P* = 0.0002). Enhancement was no longer visible at 100 Hz and was replaced by a slow decline in response amplitude, with significant difference in amplitude being visible between the first and the fifth stimulation (Fischer's PLSD, *P* = 0.02). Most notably, differences between CPT and control conditions weakened such that amplitudes no longer differed from the third stimulation at 25 Hz and from the second stimulation at 50 and 100 Hz (Fischer's PLSD, *P *>* *0.05).

These results show that, in addition to dose effect visible with the first pulse, adenosine had two further actions on signal modulation during the stimulation trains: first, the relative enhancement during the stimulation trains was stronger with higher adenosine concentration; second, the frequency at which modulation was the strongest seemed to shift toward higher values with the increase in extracellular adenosine concentration. The most noticeable consequence of these effects was that the inhibition induced by adenosine lost a great part of its influence during high‐frequency stimulation.

**Figure 5:**
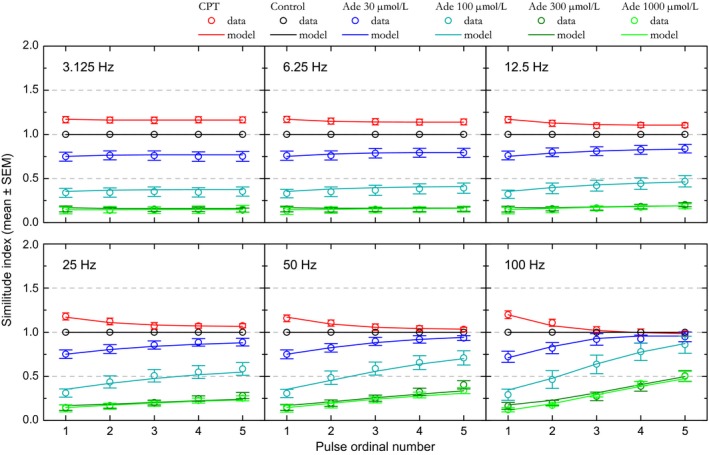
Changes in “similitude index” indicate that short‐term synaptic plasticity counteracts adenosine inhibition at high stimulation frequency. The similitude index corresponds, for a stimulus of order *n*, to the ratio of N‐wave amplitude in a given condition (adenosine or CPT) divided by the control N‐wave amplitude. Similitude indices associated with each condition are plotted as a function of stimulation pulse rank and stimulation frequency (3.125–100 Hz). Colored dots indicate the means for the different tests (CPT and adenosine at 30, 100, 300, and 1000 *μ*mol/L). Error bars represent the SEM. Continuous lines represent the mean similitude index calculated from values predicted by the STP model. SEM values for model predictions were similar to those for experimental data and are not shown for alleviating the figure.

Hence the initial loss in response amplitude seemed to be compensated by STP. This frequency‐dependent counterbalance is further illustrated in Figure 5, which represents the “similitude index” with respect to adenosine concentration and stimulation frequency. The similitude index, *SI*, is calculated as the response amplitude obtained with a stimulus of rank *n* in one condition (*cond)* divided by the response amplitude obtained with the *same* stimulus rank in the control (*cont*) condition: SI_*n*_ = *A*_cond,n_/*A*_cont,n_. In the presence of adenosine, the SI remained at a steady level below unity for all stimuli at low frequency (3.125 and 6.25 Hz); conversely, in the presence of CPT, the SI remained at a constant value above unity for all stimuli at low frequency (3.125 and 6.25 Hz). For frequencies between 12.5 Hz and 50 Hz, the SI increased during the stimulation train in the presence of adenosine, while it tended to decrease in the presence of CPT. In the presence of CPT, the SI did not differ significantly from unity with pulses 3–5 at 50 Hz and at 100 Hz. Likewise, in the presence of adenosine 30 *μ*mol/L, the SI did not differ significantly from unity with pulses 3–5 at 100 Hz. Finally, the SI value with the last stimulus at 100 Hz in adenosine 100 *μ*mol/L did not differ significantly from unity. At 100 Hz, the effects of adenosine (30–100 *μ*mol/L) or of adenosine receptor blockade were fully compensated by STP in the middle or at the end of the stimulation train.

### Short‐term plasticity model

STP is a combination of facilitation and depression mechanisms. These mechanisms possess different time constants that determine the speed of recovery to the steady state. Depending on stimulation frequency, these phenomenological rules directly influence relative amplitude evolution. To quantify the effect of adenosine on STP, we fitted the data with the model (see Methods) we previously used to explore the influence of calcium on STP in piriform cortex (Gleizes et al. 2017).

To fit the present data, we used two variants of the model: the first one with facilitation only, the second with facilitation and either one or two depression mechanisms. As our stimulation protocol was limited to five pulses per stimulation train, our STP model could rest only on these three mechanisms – several additional STP mechanisms have been characterized, but their activation requires a high number of stimuli (see Discussion). Fittings were made at once on experimental datasets consisting in one control and one CPT condition, or in one control and 1–3 adenosine conditions, with *E*, the global synaptic efficacy, shared across conditions.

As highlighted in the examples in Figures 2C and 3C, the STP model reproduced the observed STP behaviors in both control conditions as well as in CPT and adenosine 100 *μ*mol/L conditions (solid lines for model compared to dots in same color for observed data). In the control condition of the first example (Fig. 2C), the modeled STP rests on two mechanisms: a facilitation mechanism with a recovery time constant around 150 msec and one depression mechanisms that recovered with a time constant of 19 msec. Thus, enhancement up to 25 Hz can be explained by the dominance of a facilitation mechanism with a long time constant of recovery. For higher frequencies, the depression counteracted facilitation and led to a decrease in relative amplitude. The model fit obtained in the presence of adenosine 100 *μ*mol/L also implicated two mechanisms, facilitation with a slightly longer recovery time constant (184 msec) and depression with a shorter recovery time constant (11 msec). The main difference with the control condition was a large decrease in *U* (−78%), indicating a strong decrease in synaptic resource utilization.

The modeled STP in Figure 3C rests on three mechanisms, one facilitation mechanism and two depression mechanisms that recovered with different time constants. In the control condition, the fastest depression recovered with a time constant of 14 msec while the slowest recovered with a time constant of 100 msec. The parameter *k* displayed a high value (0.92), indicating more importance for the depression associated to the fastest recovery time constant. The recovery time constant for the facilitation mechanism was 163 msec. The data were also best fitted with the three‐mechanisms model in the CPT condition. The difference with the control situation was mostly explained by an increase by 16% in synaptic resource utilization at the first pulse, *U* (*U*_Ctrl_ = 0.575 vs. *U*_CPT_ = 0.666). A decrease in slow depression recovery time constant was also noticed (*τ*_*R*2_, 62 msec in CPT condition). The other parameter values were similar to those in control condition (*τ*_*F*_ = 167 msec; *k *=* *0.89; *τ*_*R*1_ = 12 msec).

STP has been examined in 46 of the 54 datasets used in the dose–response relationship (see above). For the remaining datasets, model optimization rested on MSE minimization. The model fit quality was evaluated with the root mean squared error (RMSE), which has the same dimension as the relative amplitude. Seven additional datasets have been excluded from further consideration because the RMSE returned by the fit was >0.1. For the remaining 39 datasets, the mean RMSE was 0.047 ± 0.003 – that is, a < 5% difference between observed and predicted data on average.

**Figure 6:**
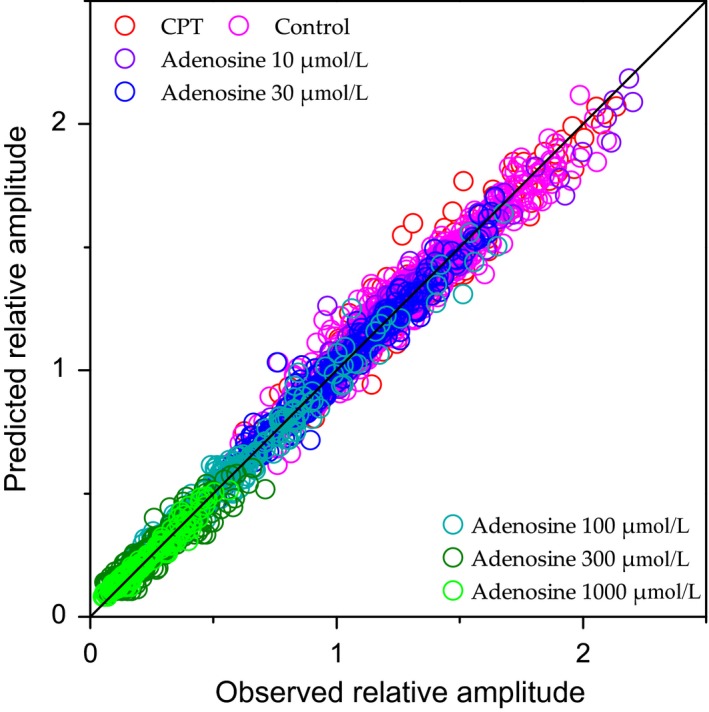
Scatterplot of predicted relative amplitudes as a function of observed relative amplitudes. Each relative response amplitude is symbolized by a color corresponding to the associated experimental condition. In total, 2620 pairs of values are represented. Perfect prediction is represented by the diagonal in black. A linear correlation was calculated to compare the model predictions to the observed values, which gave an *r²* of 0.988.

At the population level, relative amplitudes predicted by the model were averaged and are represented by solid lines in Figure 4. Mean predicted values appear to be quite similar to the mean observed values. Goodness of fit is further illustrated in Figure 6, which represents the predicted data as a function of the observed data. The alignment to the diagonal of equality and the global correlation coefficient, *r²*, of 0.988 attest for the model fit quality.

**Figure 7:**
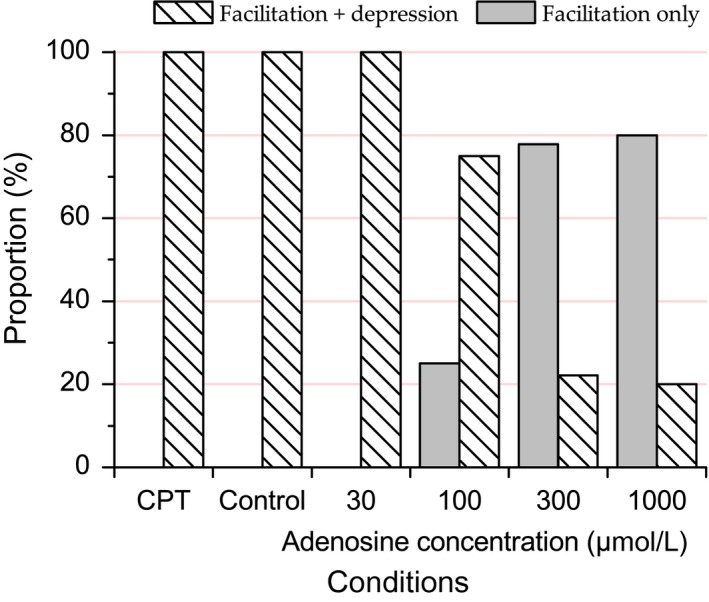
Proportion of models fitted with both depression and facilitation mechanisms or with facilitation mechanism only. Number of cases fitted with either one or two depression mechanisms did not depend on experimental conditions such that cases with either one or two depression mechanisms have been lumped together. The proportion of cases fitted with facilitation only (vs. facilitation + one or two depression mechanisms) was significantly dependent on the experimental condition and was higher with higher adenosine concentrations.

For a given experimental condition, two model variants could be used: with facilitation only, or with facilitation and at least one depression mechanism (either fast depression or both fast and slow depressions). The choice of the variant that best fitted the data rested on the MSE. The proportion of model variants that best fitted the data depended significantly on the experimental conditions (*χ* ^2^ test, *P *<* *0.0001). More precisely, as summarized in Figure 7, adequate fit of STP data for adenosine concentrations at 30 μmol/L, as well as for control and for CPT conditions, required in all cases models with at least one depression mechanism in addition to the facilitation mechanism. Yet the model without depression (facilitation only) yielded better fits with the highest adenosine concentrations (300–1000 *μ*mol/L), and the proportion of fits without depression increased with adenosine concentration: 25% (2/8 cases) with adenosine 100 *μ*mol/L, 78% (7/9) with adenosine 300 *μ*mol/L, and 80% (4/5 cases) with adenosine 1000 *μ*mol/L. On the other hand, requirement for one or two depression mechanisms did not depend significantly on experimental condition (*χ* ^2^ test, *P* = 0.14).

**Figure 8:**
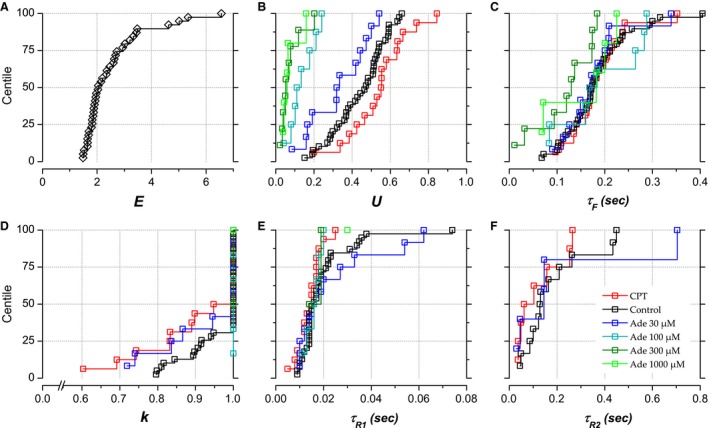
Short‐term synaptic plasticity parameters: cumulative distributions at the population level. Parameters optimized to fit the observed STP data are presented as cumulative distributions (centile plots) for data obtained in control condition (black), in CPT (red) and in adenosine 30–1000 *μ*mol/L (blue, cyan, olive, and green). The line at 50% in centile plots indicates the median of the distributions and lines at 25% and 75% delineate the interquartile range. (A) *E*, global synaptic efficacy. (B) *U*, utilization of efficacy at the first stimulation. (C) *τ*_*F*_, recovery time constant of facilitation mechanism. (D) *k*, coefficient defining the partition of synaptic resources to depression mechanisms with either fast or slow recovery (slow‐depression mechanism dismissed when *k* = 1). (E) *τ*_*R*1_, time constant of recovery for fast‐depression mechanism. (F) *τ*_*R*2_, time constant of recovery for slow depression mechanism.

Distributions of STP parameters are represented as cumulative distribution in Figure 8. *E*, as the maximal potential of synaptic transmission, is the ceiling level for response amplitude. Among the 39 datasets, *E* was distributed from 1.49 to 6.56 with a mean of 2.54 ± 0.18.

**Figure 9:**
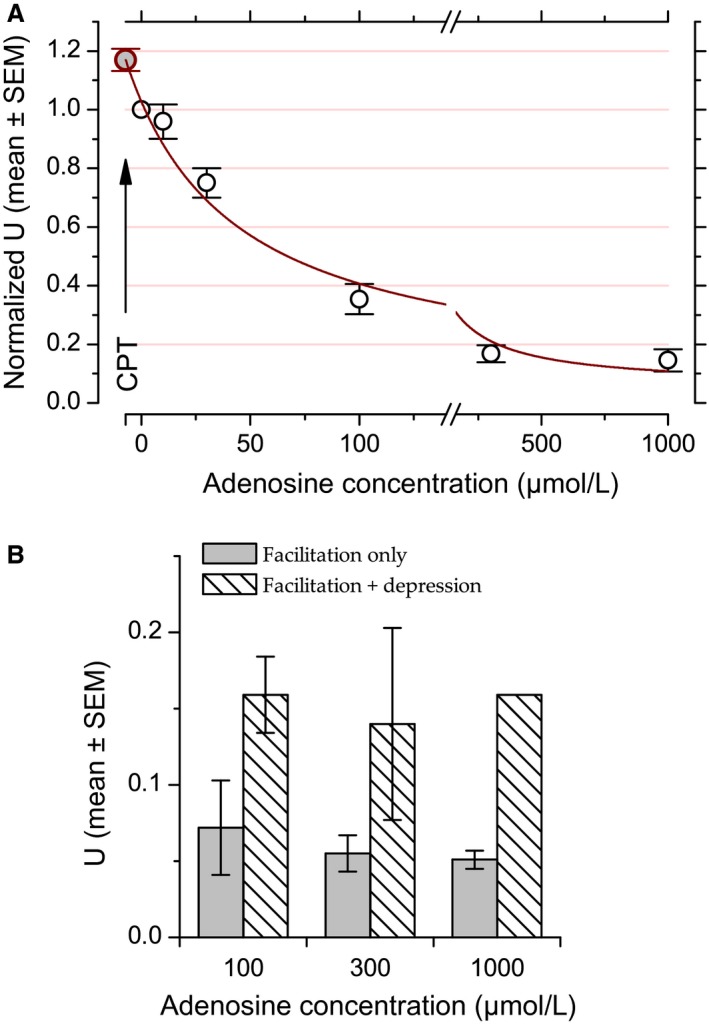
(A) Changes in model parameter *U* as a function of experimental condition fitted with Prince and Stevens (1992)'s model. Data points correspond to the mean and error bars to ±1 SEM computed after normalizing the individual *U* values to their associated control values. For enhancing data visibility, the *x*‐axis has been split and is presented with different scales before and after the break. The red line corresponds to Prince and Stevens (1992)'s model fit (weighted by variance, *R* ^2^ = 0.98). The data point corresponding to CPT condition at coordinates *c*_0_ = −7.3 *μ*mol/L and *U*_max_ = 1.17 (arrow) was added after fitting. (B) Requirement for depression in model fit is associated with higher values of *U*. Bar height represents the mean *U* value (±1 SEM) in model best fitted with (hatched bars) or without (grey bars) depression mechanisms in the presence of adenosine at 100, 300, and 1000 *μ*mol/L.

*U* can be defined as the initial probability of release. In control condition, *U* averaged 0.442 ± 0.022. As illustrated in Figure 9A, the application of CPT resulted in a significant (*P* = 0.001, paired *t*‐test) increase of *U* by 17% [10–24%] in comparison to paired control. Conversely, *U* decreased in proportion to adenosine concentration. In adenosine 30 *μ*mol/L, *U* reached a value representing 75% [65–85%] of the control value (*P* = 0.001). In adenosine 100 *μ*mol/L, *U* reached a value representing 35% [25–46%] of the control value (*P* = 0.001). In adenosine 300 *μ*mol/L, *U* reached a value representing 17% [11–23%] of the control value (*P* = 0.0002) and a value representing 15% [7–22%] of the control value (*P* = 0.001) in adenosine 1 mmol/L.

As *U* reflects the first synaptic resource utilization, it should vary in a similar way as the initial relative amplitude; in other words, its variation should reflect the dose–response effect of adenosine (Fig. 1). To go further, we fitted the changes in *U* in the presence of CPT and of increasing concentration of adenosine with Prince and Stevens (1992)'s model (see Methods) (Fig. 9A), as previously done for the relationship between adenosine concentration and synaptic response amplitude (Fig. 1C). The mean value associated to CPT condition, *U*_*max*_ (without effect of ambient adenosine on A1 receptors) allowed determining *c*_0_, the estimated endogenous adenosine concentration, at 7.3 *μ*mol/L (Fig. 9A). This value is less than that estimated with the experimental data (11.4 *μ*mol/L, Fig. 1C) but is of the same order of magnitude. The adenosine concentration required to induce a 50% decrease in *U* was estimated at 60 *μ*mol/L (Fig. 9A), close to the *IC_50_* determined from the experimental data (70 *μ*mol/L, Fig. 1C).

With adenosine concentration between 100 and 1000 *μ*mol/L, either facilitation alone, or both facilitation and depression were required to fit the experimental data (Fig. 7). We examined whether these different requirements could be related to *U*. As illustrated in Figure 9B, there was indeed a relationship between *U* values and model types that best fitted the data. For this range of adenosine concentrations, the mean *U* value was larger in cases requiring both depression and facilitation (0.14–0.16 on average) in comparison to cases requiring facilitation only (0.05–0.07 on average). The presence or absence of depression showed a highly significant dependence on *U* value (ANOVA, *P* = 0.002). These results suggest that there is a threshold value for *U* below which STF is the sole mechanism involved in STP in our five‐stimulus protocol. Examination of *U* value distribution (not illustrated) suggested that this threshold *U* value is around 0.1.

In contrast to *U*, other model parameters did not appear to be significantly affected by CPT or adenosine and their cumulative distributions all overlapped to some extent (Fig. 8). The recovery time constant for facilitation, *τ*_*F*_, presented a mean value of 183 ± 11 msec in control condition. As shown in Figure 8, the same range of values was observed in the different experimental conditions and no significant difference could be detected.

For data fitted with depression mechanisms, the parameter *k* determines the allocation of the synaptic resources into two subgroups, one that shows a fast and the other a slow recovery from depression. *k* is equal to 1 when only the fast depression mechanism is required for fitting the data, which was the case for about two‐thirds of the cases in control condition and half the cases in CPT. At the population level, *k* was not significantly affected by CPT or adenosine (Fig. 8, *P* > 0.05 for all paired comparisons). In control condition, *k* ranged between 0.796 and 1 with a mean of 0.961 ± 0.011.

Time constants for fast‐ and slow‐depression recoveries also seemed to be independent of the tested conditions (Fig. 8; *P *>* *0.05 for all comparisons). In control condition, the recovery time constant for the fast depression mechanism averaged 19 ± 2 msec. For the slow depression mechanism, the recovery time constant averaged 181 ± 39 msec. (Although their ranges were similar, the recovery time constants for slow depression and for facilitation were not significantly correlated: *P* = 0.06, *r* ^2^ = 0.14; not illustrated.)

**Figure 10:**
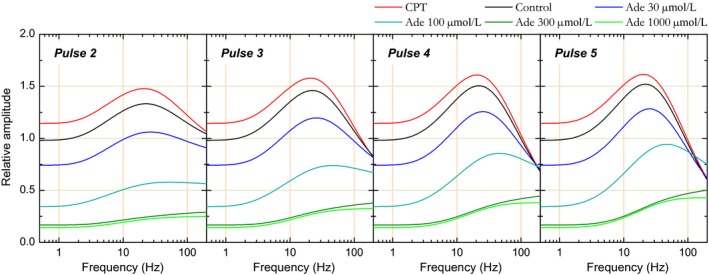
Simulation of relative response amplitude as a function of stimulation frequency reconstructed from short‐term plasticity model parameters in the different conditions and for each consecutive stimulation pulse. Simulations were first produced for each experimental condition and each stimulation pulse using model parameters issued from each individual case. Simulations pertaining to the same stimulation pulse and to the same experimental condition were then averaged and the corresponding curves are presented in the figure: panels correspond to pulses 2–5 and the different lines in each panel to the different experimental conditions (CPT, control, and adenosine 30–1000 *μ*mol/L).

We next used the parameters determined from the model to obtain a fine‐grained image of STP and of the effect of adenosine on STP at the LOT‐Layer 1 synapse. For this purpose, curves were generated over a continuum of frequencies between 0.1 and 1000 Hz for each dataset using the model parameters associated with this dataset. Curves representing relative amplitude as a function of stimulation frequency were generated for each experimental condition and for each consecutive stimulation pulse. Figure 10 shows the curves for each pulse after averaging for each experimental condition. At low frequency (≤1 Hz), differences in relative amplitude reprise the inhibitory action of exogenous and endogenous adenosine. As stimulation frequency increases, relative amplitude progressively increases up to reaching a maximal value near 20–40 Hz in control, CPT or adenosine 30–100 *μ*mol/L (see below) while no such peak was reached in adenosine 300–1000 *μ*mol/L. The maximal response enhancement was followed by a progressive decline of relative response amplitude in control, CPT or adenosine 30–100 *μ*mol/L. In control, CPT and adenosine 30 *μ*mol/L, but not in adenosine 100 *μ*mol/L, response decline brought relative amplitude to values below those obtained at ≤1 Hz at 100–200 Hz. Most noticeable is the observation that the curves come closer and closer as frequency increases; depending on pulse number, differences in response amplitude ultimately disappear between 100 and 200 Hz in all experimental conditions except those with the highest adenosine concentrations (300 and 1000 *μ*mol/L).

**Figure 11:**
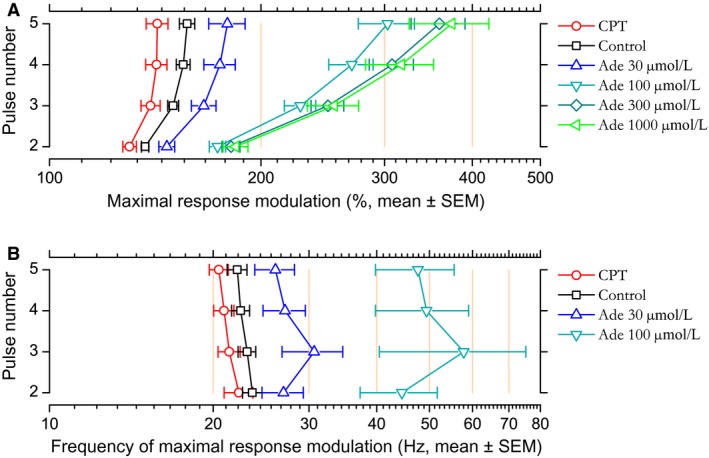
(A) Maximal response modulation in CPT, control, and different adenosine concentrations. Maximal response modulation was extracted from individual simulation of STP calculated using parameters of the model fitted to each individual case. Dots correspond to the means calculated for each consecutive stimulating pulse and error bar represent the SEM. (B) Frequency at which maximal response modulation occurred (mean ± SEM). This frequency could not be determined for simulations that lacked a peak followed by a decline for frequencies ≤200 Hz. As a consequence, reduced sample sizes precluded showing frequency data for adenosine 300 and 1000 *μ*mol/L.

Two values were extracted from each individual fine‐grained simulation: the maximal response modulation, which corresponds to the maximal response amplitude normalized by the response amplitude obtained with the first pulse in the same experimental condition, and the frequency at which the maximal modulation was achieved. The frequency of maximal response enhancement was undetermined for curves that lack response decline (this concerned 2/8 simulations in adenosine 100 *μ*mol/L, 7/9 simulations in adenosine 300 *μ*mol/L and 4/5 simulations in adenosine 1000 *μ*mol/L); in these cases, the maximal modulation was taken as the value reached at 200 Hz. Figure 11 shows the means of these two measurements.

In the control condition, the maximal response modulation was between +37% with the second stimulation of the train and +54% with the fifth stimulation of the train (Fig. 11A). Maximal enhancement occurred within the beta‐band, at frequencies between 23.6 Hz (second stimulation) and 22.2 Hz (fifth stimulation) (Fig. 11B).

In the presence of CPT, the maximal response modulation was significantly less than in the control condition (paired *t*‐test, *P* ≤ 0.001) and reached values between +30% (pulse 2) and +42% (pulses 4 and 5, Fig. 11A). Maximal enhancement still occurred in the beta‐band, at a slightly but significantly lower frequency than in control condition (*P* ≤ 0.03): between 22.3 (second stimulation) and 20.5 Hz (fifth stimulation). Thus, in the absence of endogenous adenosine, response modulation was less than in the control condition and its maximum was reached at a slightly lower frequency.

The converse was observed in the presence of adenosine 30 *μ*mol/L: maximal modulation was significantly (*P* ≤ 0.005) larger than in control condition (+47% with second stimulation to +79% with fifth pulse, Fig. 11A) and the frequency at which maximal enhancement occurred was significantly (*P* ≤ 0.03) higher than in control condition, occurring in the upper beta‐band (26–31 Hz, Fig. 11B). This figure was further accentuated in adenosine 100 *μ*mol/L (Fig. 11A and B): maximal response modulation reached values between +73 and +203% (*P* ≤ 0.0003) and the frequency at which maximal enhancement took place was shifted toward the gamma‐band (44–58 Hz, *P* ≤ 0.05 except pulse 3: *P* = 0.07). Response modulation was much larger in adenosine 300 and 1000 *μ*mol/L in comparison to control (*P* ≤ 0.0001 and ≤0.02 respectively). Response modulation ranged between +81% and +259% from the second to the fifth pulse in adenosine 300 *μ*mol/L, and between +84% and +274% in adenosine 1000 *μ*mol/L (Fig. 11A).

Overall, these results show that the range of response amplitudes progressively increased as a function of adenosine concentration and that the frequency at which maximal enhancement occurred also increased in parallel. Through its action on STP mechanisms, adenosine attenuated signal transmission at low frequency, but this attenuation progressively relaxed as frequencies reached the beta and gamma bands, and vanished almost fully for signals transmitted at frequencies ≥100 Hz.

## DISCUSSION

Our experiments and model allowed determining which mechanisms of STP at the LOT‐layer 1a synapse were affected by extracellular adenosine. Globally, increasing extracellular adenosine concentration reduced the impact of depression on synaptic transmission. In parallel, elevating adenosine concentration shifted the maximal signal enhancement toward higher frequencies. The model suggests that differences in STP behaviors were mostly explained by two phenomena: (1) a decrease in initial neurotransmitter release probability (*U*) with increased adenosine concentration and (2) a reduced requirement for depression mechanisms with increased adenosine concentration. The consequence was that, despite the initial inhibition induced by adenosine (decrease in *U*), changes in STP dynamics largely compensated for the initial reduction in response amplitude, especially for signals transmitted at high frequency. This way, adenosine could be metaphorically envisioned as a high‐pass filter, efficiently inhibiting synaptic transmission at low frequency but letting through high‐frequency signals.

### Dose–response relationship

Activation of G protein‐coupled adenosine receptors in the brain can exert either facilitatory (A2_A_, A2_B_ receptors) or inhibitory action (A1 receptors) (Cunha 2001; Dunwiddie and Masino 2001). Inhibitory effect of adenosine has been established long ago in various structures of the nervous system including the neuromuscular junction (Ginsborg and Hirst 1972), neocortex (Phillis et al. 1975), hippocampus (Schubert and Mitzdorf 1979; Dunwiddie and Hoffer 1980), and piriform cortex (Kuroda et al. 1976; Scholfield 1978). The inhibitory action of adenosine rests on both pre‐ and postsynaptic mechanisms: the postsynaptic mechanism is attributed to an increase in potassium conductance (e.g., Greene and Haas 1985; McCormick and Williamson 1989; Lüscher et al. 1997); the presynaptic mechanism corresponds to a reduction of calcium conductances in axon terminals (e.g., Hamilton and Smith 1991; Wu and Saggau 1994; Wheeler et al. 1994; Emptage et al. 2001), which leads to a reduction in neurotransmitter release.

In piriform cortex, the inhibitory action of adenosine at the LOT‐layer 1a synapse has been attributed to A1 receptor activation (Collins and Anson 1985; McCabe and Scholfield 1985; Yang et al. 2007). In the first part of this study, we examined synaptic response reduction as a function of adenosine concentration at the LOT‐layer 1a synapse. We fitted the resulting dose–response relationship with Prince and Stevens (1992)'s model, which allowed extrapolating an *IC_50_* of 70 *μ*mol/L (Fig. 1C). This value is in between those reported in previous piriform cortex studies: McCabe and Scholfield (1985) and Yang et al. (2007) reported *IC_50_*s of the order of 7–8 *μ*mol/L while at the other extreme Collins and Anson (1985) reported an *IC_50_* of 139 *μ*mol/L. Several factors may explain these discrepancies. In particular, powerful adenosine uptake (through ENTs) and adenosine degradation (by adenosine deaminase) mechanisms are capable of strongly reducing bath‐applied adenosine concentration as adenosine diffuses through the brain tissue; as a result of this reduction, Dunwiddie and Diao (1994) estimated that, in hippocampus, the “real” *IC_50_* for adenosine action was between 0.60 and 0.76 *μ*mol/L only. Any environmental factor that affects adenosine diffusion, uptake, and degradation would therefore affect the apparent *IC_50_*. For example, it has been shown that the activity of adenosine transporters is strongly reduced when experiments are conducted at room temperature (Dunwiddie and Diao 2000).

Response amplitude increased by about 20% in the presence of CPT, a selective A1 receptor antagonist (Bruns et al. 1986). This confirms that endogenous adenosine generates a sustained inhibitory tone at the LOT‐layer 1a synapse through A1 receptors. In piriform cortex, a + 15% increase in postsynaptic response amplitude has previously been reported by McCabe and Scholfield (1985) after blocking endogenous adenosine intracellular signaling pathway. Yang et al. (2007), on the other hand, reported a + 84% increase in postsynaptic response amplitude after blocking ambient adenosine action with 1,3‐dipropyl‐8‐cyclopentylxanthine (DPCPX), another A1 receptor antagonist (Martinson et al. 1987). Using Prince and Stevens (1992)'s model (Fig. 1C), we could extrapolate that the endogenous adenosine concentration was equivalent to 11 *μ*mol/L of bath‐applied adenosine. The same value was obtained by Prince and Stevens (1992) in the *dentate gyrus* of the hippocampus. Using the same approach, Kerr et al. (2013) extrapolated the ambient adenosine concentration to be equivalent to 23 *μ*mol/L of bath‐applied adenosine in layer 5 of neocortex. A larger value (30 *μ*mol/L) was reported by Yang et al. (2007) in posterior piriform cortex at room temperature. For the reasons given above, the “real” basal endogenous adenosine concentration is likely to be much lower. In hippocampus in vitro, Dunwiddie and Diao (1994) estimated that this concentration would be around 0.14–0.20 *μ*mol/L while direct measurements in various brain regions in vivo returned values between 0.05 and 0.3 *μ*mol/L (e.g., Ballarín et al. 1991; Porkka‐Heiskanen et al. 2000; Bjerring et al. 2015).

In our experiments, postsynaptic response amplitude was not significantly affected by ZM 241385, a specific A2_A_ receptor antagonist (Poucher et al. 1995). This result might appear unexpected as recent studies showed that A2_A_ receptor mRNA is expressed in both mitral and tufted cells (Wang et al. 2017; Rotermund et al. 2018). One possible reason for the lack of action of ZM 241385 is the different affinity of adenosine for A2_A_ and A1 receptors (Correia‐de‐Sá and Ribeiro 1996; Fredholm et al. 2001). In particular, Fredholm et al. (2001) showed that, in transfected cells, the *EC*_50_ for adenosine was more than twice lower for A1 receptor compared to A2_A_ receptors. It is thus possible that the ambient adenosine level in our experimental conditions was just high enough for activating A1 receptors, but too low to have a measurable action on A2_A_ receptors.

A question that naturally arises is the relevance of the adenosine concentrations we used, with respect to adenosine concentration changes observed in physiological or pathological conditions. A rough correspondence between adenosine concentrations used in this study and changes in adenosine level measured in various experimental conditions can be proposed. Using Prince and Stevens’ model, we estimated that the ambient adenosine concentration was equivalent to ≈10 *μ*mol/L of bath‐applied adenosine (Fig. 1C). Several studies reported changes in adenosine concentration in various physiological and pathological contexts. For instance, in hippocampus in vitro a 3‐min‐long stimulation train at 5 Hz increases extracellular adenosine level by a factor of ≈5 relative to ambient adenosine concentration (Cunha et al. 1996) while a 5‐min‐long stimulation train at 10 Hz produces an increase by a factor of 10 (Lloyd et al. 1993). With respect to our endogenous adenosine concentration estimate, the ×5 increase would correspond to a bath‐applied concentration of 50 *μ*mol/L, and the ×10 increase to a bath‐applied concentration of 100 *μ*mol/L. Even larger increases of adenosine levels have been observed in pathological conditions. For example, During and Spencer (1992) reported that, during seizures, adenosine level was increased by a factor between 7 and 31 in the hippocampus of epileptic patients, which would correspond to up to ≈300 *μ*mol/L of bath‐applied adenosine. Finally, short (4–5 min) and long‐lasting (10 min) ischemic episodes have been reported to increase adenosine concentrations by a factor of 35 (Kobayashi et al. 1998) and 70 (Onodera et al. 1986), respectively. These factors would correspond to bath‐applied adenosine concentrations of 350 and 700 *μ*mol/L, respectively. So we can tentatively conclude that the range of adenosine concentrations we used matches with the changes in adenosine concentration encountered both in physiological and pathological conditions.

### Effect of adenosine on short‐term plasticity

The main purpose of this study was to examine the effect of adenosine on short‐term synaptic plasticity at the LOT‐layer 1a synapse of the adult mouse anterior piriform cortex in vitro using environmental conditions (temperature, ACSF ionic concentrations) as close as possible to those that prevail in vivo. For this purpose, we used 5‐pulse trains of stimuli emitted at frequencies between 3.125 and 100 Hz. This frequency range allowed approximating the influence of oscillations identified in vivo, in particular those observed in the main input to the piriform cortex, the olfactory bulb: respiratory rhythm, *β* and *γ* fluctuations.

In comparison to several other connections, one specific feature of the LOT‐layer 1a synapse is a noticeable enhancement of response amplitude during high‐frequency stimulation (e.g., Maclean et al. 1957; Richards 1972; Suzuki and Bekkers 2011; Gleizes et al. 2017). In agreement with our previous study (Gleizes et al. 2017), this enhancement was barely visible with the lowest frequency tested (3.125 Hz), but was noticeable at 12.5 Hz, was maximal at 25 Hz, and still substantial at 50 Hz. It was barely visible at the highest frequency tested (100 Hz) and was eventually replaced by a response decline (Fig. 4). As described in Gleizes et al. (2017), response enhancement at *β* and *γ* frequencies would have been missed, had we used the environmental factors that are traditionally used in in vitro studies – in particular extracellular calcium concentration.

As far as piriform cortex is concerned, only two studies reported on the action of adenosine on short‐term synaptic plasticity (Okada and Saito 1979; Yang et al. 2007). In both studies, examination of STP was limited to the analysis of paired‐pulse ratios (PPR). Both studies reported that the PPR increased in the presence of bath‐applied adenosine. Yang et al. (2007) also reported a complementary decrease in the PPR when blocking ambient adenosine action on A1 receptors with DPCPX. Increase in PPR, attributed to the action of adenosine on presynaptic A1 receptors, has also been reported in many other brain regions (e.g., neocortex: Murakoshi et al. 2001; Fontanez and Porter 2006; Bannon et al. 2014; Qi et al. 2017; hippocampus: Debanne et al. 1996; Moore et al. 2003; hypothalamus: Oliet and Poulain 1999; calyx of Held: Wong et al. 2006).

To our knowledge, the effect of adenosine on STP examined with trains of several stimulating pulses has not been studied in the piriform cortex. The general tendency with series of stimulating pulses in other brain regions is a reduction of apparent STD and/or a strengthening of apparent STF in the presence of adenosine (e.g., neuromuscular junction: Redman and Silinsky 1994; neocortex: Varela et al. 1997; Kerr et al. 2013; Qi et al. 2017; hippocampus: Pananceau et al. 1998). The predominant effect of adenosine in our study was to reinforce the enhancement of response amplitude in comparison to that in control condition while blocking endogenous adenosine action had the opposite effect (Figs. 4, 10, 11). The strengthening of response enhancement depended on both stimulation frequency, pulse ordinal number and adenosine concentration (Figs. 4, 10, 11). For example, relative to the response amplitude obtained with the first stimulation in the same experimental condition, response amplitude with the fifth stimulation was 40% larger in CPT, about 60% larger in control, about 80% larger in adenosine 30 *μ*mol/L, and three times larger in adenosine 100 *μ*mol/L (Fig. 11). Moreover, the frequency at which maximal response enhancement occurred increased with increase in adenosine concentration, from about 20–22 Hz in CPT to 22–24 Hz in control, 26–30 Hz in adenosine 30 *μ*mol/L and ≥ 45 Hz with adenosine 100 *μ*mol/L (Fig. 11).

To further examine the effects of adenosine concentration and stimulation frequencies, response amplitudes were compared on a pulse by pulse basis with those in control condition. The “similitude index” thus computed (Fig. 5) revealed that responses obtained in CPT and in the presence of adenosine remained at a steady level when evoked at 3.125 and 6.25 Hz. Yet, at 12.5 Hz and higher frequencies, the response amplitude difference with respect to control response amplitude decreased as the stimulation train progressed. This progressive reduction in amplitude difference was stronger with higher stimulation frequency, to the extent that response amplitude difference eventually vanished: in CPT during the 25, 50, and 100 Hz stimulation train, and in adenosine at 30 and 100 *μ*mol/L at the end of the 100 Hz stimulation train. Yet a complete suppression of response amplitude difference was not achieved with adenosine at 300 and 1000 *μ*mol/L. For adenosine concentration ≤ 100 *μ*mol/L, these results suggest that, in contrast to a postsynaptic inhibitory mechanism that would reduce response amplitude independently from afferent input frequency, the presynaptic inhibition mediated by adenosine A1 receptors effectively suppresses low‐frequency inputs but let go high‐frequency inputs, hence acting in a manner analogous to that of a high‐pass filter.

### Mechanisms involved in the action of adenosine on short‐term synaptic plasticity

The results we obtained implicate two opposite actions of adenosine: first a reduction in response amplitude at 0.5 Hz, that remained unchanged at 3.125 and 6.25 Hz; second, a reinforcement of response enhancement, which was most visible with high‐stimulation frequencies. The phenomenological model of STP we applied to the data allowed determining with mechanisms could account for these effects of adenosine.

Short‐term synaptic plasticity rests on multiple mechanisms characterized by different time courses (e.g., Curtis and Eccles 1960; Richards 1972; Zucker and Regehr 2002; Fioravante and Regehr 2011; Holohean and Magleby 2011; Hennig 2013; de Jong and Fioravante 2014). Thus, on the basis of their dynamics, up to three distinct STF mechanisms have been distinguished: facilitation proper, with a time course in the hundreds of msec range, and two mechanisms whose influences persist for seconds to minutes: augmentation and post‐tetanic potentiation. Yet, in contrast to facilitation that can already be demonstrated with pairs of stimuli, augmentation and post‐tetanic potentiation become visible only after long‐lasting repetitive stimulation. As for STF, several types of STD have been evidenced, with characteristic time courses: a fast STD recovering within a few tens of msec, an intermediate mechanism recovering within few hundreds of msec, and a slow depression lasting over several seconds. However, as for augmentation and post‐tetanic potentiation, large number of stimuli is required to induce the slow depression mechanism. Interestingly, studies showed that this slow depression mechanism could depend on an increase in endogenous adenosine production during the stimulation trains (Mitchell et al. 1993; Lovinger and Choi 1995; Oliet and Poulain 1999; Brager and Thompson 2003; Wong et al. 2006; Lovatt et al. 2012; Wall and Dale 2013).

As we used only few pulses per stimulation train, our STP model could rest only on the three fastest STP mechanisms: a facilitation mechanism and two depression mechanisms with fast and slow recoveries – our slow mechanism would actually correspond to the intermediate mechanism mentioned in the preceding paragraph. In our control condition, facilitation, with a mean recovery time constant of 183 msec, accounted for response enhancement at frequencies ≥ 3 Hz. The slow depression, with a mean recovery time constant of about 180 msec, could eventually counterbalance the facilitation, although it was required in only about half the fits. The relatively weak influence of the slow‐depression mechanism likely results from our in vivo‐like experimental conditions. It has been shown to play a more prominent role when the ACSF contains higher calcium concentration (Dittman and Regehr 1998; Gleizes et al. 2017). The fast depression, with a mean recovery time constant of about 20 msec, could explain the response decline observed at high‐stimulation frequency (≥ 25 Hz).

The main effect of manipulating endogenous and exogenous adenosine concentrations was a significant change for the first‐synaptic resource utilization, *U*. *U* increased in the presence of CPT and decreased in proportion to the concentration of bath‐applied adenosine. This readily explains changes in response amplitude at the first stimulation pulse, as it is solely determined by *U*. The changes in *U*, extrapolated from the STP model fit (Fig. 9A), were actually very close to the changes in response amplitude directly measured in the experimental data at low frequency (Fig. 1C). Kerr et al. (2013), who analysed STP between neocortical layer 5 pyramidal cells using Tsodyks–Markram's model, also showed that the main effect of endogenous and exogenous adenosine was to reduce the initial release probability.

None of the other model parameters showed significant changes with changes in adenosine concentration. This is at variance with the results obtained in our previous study (Gleizes et al. 2017), where we found that changing extracellular calcium concentration impacted on two additional parameters in addition to *U*: the time constant of facilitation, *τ*_*F*_, which increased when calcium concentration was doubled, and the sharing of synaptic resources, *k*, which decreased when calcium concentration was doubled.

In this study, we nevertheless noticed another consequence of increasing adenosine concentration: the number of dynamic components required for fitting the data progressively decreased (Fig. 7). With CPT, in control, and with adenosine at 30 *μ*mol/L, all model fits required both depression and facilitation mechanisms. With adenosine at 100 *μ*mol/L, a couple cases could be adequately fitted with only the facilitation mechanism. With adenosine at 300 and 1000 *μ*mol/L, the facilitation mechanism alone was sufficient to fit the majority (≈80%) of cases.

These results may be explained by the presence of a “threshold” below which the use of synaptic resources (low values of *U* in high adenosine concentration) is too low to be significantly affected by depression mechanisms, such that only facilitation takes place (Fig. 9B). Our model does not include such a threshold but future development could make it explicit.

Another limitation of our study is that it was based on LFP recordings. We used low‐intensity electrical stimulation in order to examine STP of monosynaptic excitatory responses. Yet STP differs markedly between the different categories of cells of the piriform cortex, with stronger response enhancement in superficial pyramidal cells and neurogliaform cells in comparison to semilunar cells and inhibitory horizontal cells (Suzuki and Bekkers 2006, 2010, 2011). As LFP recording averaged the signals arising from these different cell types, examination of single axon EPSPs would help further refine our understanding of the mechanisms underlying the frequency dependence of adenosine action.

## Conclusions

Our study suggests that signals transmitted at high frequency, corresponding in particular to gamma and high‐gamma oscillations, are less attenuated by adenosine than signals transmitted through lower‐frequency oscillatory regimes. The meaning of these results in the context of odor coding can be speculated as follows:

Occurrence of beta and gamma oscillations are hallmarks of odor processing in the olfactory bulb (e.g., Adrian 1950; Chapman et al. 1998; Buonviso et al. 2003; Neville and Haberly 2003; Martin et al. 2004; Beshel et al. 2007; Fourcaud‐Trocmé et al. 2014). It is to be recalled however that neuronal spiking rates in the olfactory bulb are not confined to these two frequency bands: first, it has been shown that the majority of the olfactory bulb neurons can fire bursts of action potentials, with an intraburst frequency in excess of 100 Hz (Lestienne et al. 1999; Leng et al. 2014); second, odorant stimulation can increase the firing rate of olfactory bulb neurons with transients that are ≥100 Hz (e.g., Buonviso et al. 2003; Cury and Uchida 2010; Shusterman et al. 2011). As spike firing tends to be phase‐locked with beta and gamma oscillations (e.g., Gray and Skinner 1988; Eeckman and Freeman 1990; Kashiwadani et al. 1999; Cenier et al. 2009; Fourcaud‐Trocmé et al. 2014), these rhythms are transferred to the piriform cortex where both beta and gamma oscillations have been shown to occur upon odorant stimulation (e.g., Chapman et al. 1998; Neville and Haberly 2003; Litaudon et al. 2008; Poo and Isaacson 2009). That odor‐induced oscillations in piriform cortex depend on olfactory bulb input has been demonstrated through lesion experiments (Neville and Haberly 2003). We can hypothesize that, when ambient adenosine levels are low (as in our control or CPT conditions), transmission of beta oscillations will be favored as STP mechanisms induce the strongest suppression for frequencies around and above 100 Hz and the largest response enhancement in the beta frequency band. Yet ambient adenosine levels might increase, for example after sustained afferent activity. In this case, adenosine would promote adaptation by attenuating olfactory bulb inputs that are transmitted at low frequency, but would spare those signals that are transmitted within the gamma frequency band and above (Figs. 5, 10). Finally, adenosine levels might be dramatically increased in pathological conditions. In these cases, most of the olfactory bulb input would be strongly reduced, except for a relative sparing for very‐high frequency input (≥100 Hz) for which depression is replaced by facilitation. Beyond peripheral inputs, oscillations are further sustained through interactions involving networks of interconnected inhibitory and excitatory neurons (Eeckman and Freeman 1990; Poo and Isaacson 2009). How adenosine influences network interactions at this higher level of complexity warrants further study.

## Conflict of Interest

The authors declare that they have no conflict of interest.
